# Successful Treatment of Hemifacial Myokymia and Dystonia Associated to Linear Scleroderma “En Coup de Sabre” with Repeated Botox Injections

**DOI:** 10.1155/2012/691314

**Published:** 2012-08-08

**Authors:** Carlos A. Cañas, Jorge L. Orozco, Andrea Caicedo Paredes, Fabio Bonilla-Abadía

**Affiliations:** ^1^Department of Rheumatology, Fundación Valle del Lili, ICESI University, Cali, Colombia; ^2^Departament of Neurology, Fundación Valle del Lili, Cali, Colombia; ^3^Department of Internal Medicine, Fundación Valle del Lili, Cali, Colombia

## Abstract

Linear scleroderma “en coup de sabre” (LSCS) is a form of localized scleroderma presents as band-like sclerotic lesions of the frontoparietal area. It has been reported in association with diverse neurological manifestations like seizures, migraine, neuromyotonia, dystonia and abnormalities in MRI and CT studies as cerebral atrophy, white matter lesions, intraparenchymal calcification, meningeocortical alterations, and skull atrophy. We describe a patient with LSCS associated with two abnormal movements: permanent myokimia of right masseter muscle and recurrent spasmodic retraction of right cigomatic and depressor labii inferioris muscles. He was initially treated with methotrexate and steroids without response, so later on he underwent repeated Botox injections with remarkable improvement.

## 1. Introduction

Linear scleroderma “en coup de sabre” (LSCS) is a form of localized scleroderma presents as band-like sclerotic lesions with more or less marked skin discoloration of the frontoparietal area. It has been reported in association with diverse neurobiological manifestations [1]. The pathogenesis is unknown; however, the neuropathological findings, increase of IgG intrathecal, and the response to immunosuppressive treatment in some cases, provide evidence for a potential autoimmune process [2]. We describe an adult male patient with LSCS who presented right facial myokymia and dystonia with no response to steroid and methotrexate, and a favorable response to local Botox injections.

## 2. Case Report

**Figure 1:**
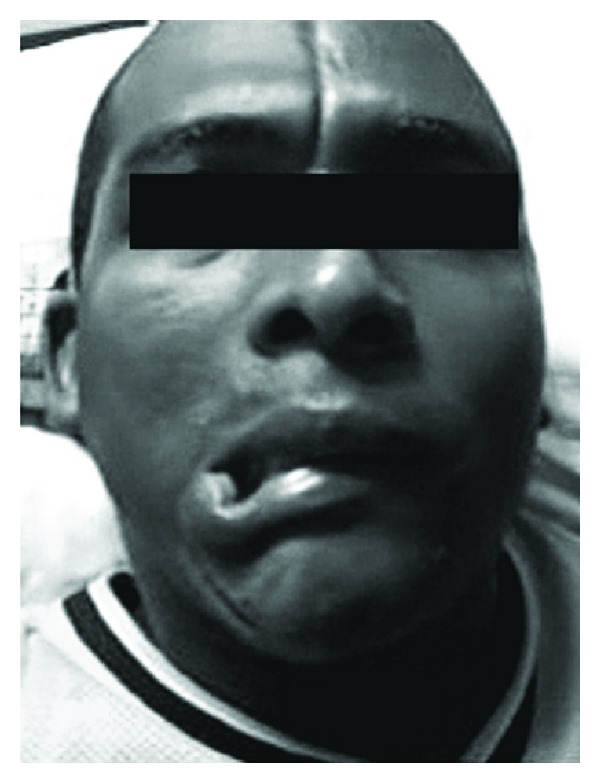
Atrophic and hyperpigmented band of sclerotic skin over frontal scalp and retraction of right cigomatic and depressor labii inferioris muscles.

A 31-year-old black man was admitted by one-year history of appearance of a hyperpigmented frontal line associated to abnormal movements in the right cheek muscles. These symptoms became more frequent and limited his daily activities. No other cutaneous, osteoarticular, cardiopulmonary and systemic symptoms was referred. General examination revealed an atrophic and hyperpigmented band of sclerotic skin over frontal scalp (Figure 1). There was no facial atrophy, eyelash or eyebrow loss. The neurologic examination was remarkable only for myokymia of right masseter muscle and recurrent dystonic retraction of right cigomatic and depressor labii inferioris muscles (Figure 1).

Laboratory exams reported positive antinuclear antibody titers 1 : 40 with homogenous pattern, negative rheumatoid factor, liver and renal function were normal, leukocyte count, electrolytes, thyroid stimulant hormone, and glucose were within normal limits. The brain Magnetic Resonance Image (MRI) was normal and the masseter muscle electromyography showed polyphasic motor units of large size and duration. A diagnosis of LSCS associated to local neuromiological manifestations was made based on the skin and muscle changes. A 3-day course of high-dose IV methylprednisolone (500 mg qd) was administered and then a regimen of prednisone 25 mg qd was continued. The patient reported persistent symptoms a month later. Methotrexate was added at a dose of 15 mg/week for six months without response. Then, we decided to start the application of local Botox injections simultaneously into different areas of right muscles engaging the frontalis, procerus, glabellar, temporalis, major and minor cigomatic, masseter, orbicularis oris, depressor angle of the mouth, chin, platysma, and contralateral compensatory points with successful control of abnormal movements. The systemic treatment was withdrawn. Repeated local application of Botox has been necessary, because of recurrent symptoms. Main outcome measures were determined by the severity and duration of response and our patient had a progressive response evidenced by a decrease of 95% in the frequency of episodes and the presence of very mild occasional dystonic contractions. These injections are applied every six months.

## 3. Discussion

Several neurological manifestations as complex partial seizures, migraine, neuromyotonia, and dystonia and abnormalities in MRI and CT studies as cerebral atrophy, white matter lesions, intraparenchymal calcification, meningeocortical alterations, and skull atrophy [3], have been associated to LSCS. The imaging findings may be present in absence of neurologic disease and normal imaging can be seen despite of the presence of neurologic disease. We report in this paper a patient with hemifacial myokymia and dystonia and a normal brain imaging. 

Botox injection has demonstrated utility in blepharospasm, hemifacial spasm, cervical, oromandibular, laryngeal, hand dystonia and spasticity caused by trauma, cerebral palsy, vascular accident and multiple sclerosis [4]. Few cases are reported in abnormal movement associated with LSCS, which included hemimasticatory spasm [5].

We describe a patient with LSCS associated with two abnormal movements: permanent myokimia of right masseter muscle and recurrent spasmodic retraction of right cigomatic and depressor labii inferioris muscles, a rare form of presentation. He was initially treated with methotrexate and steroids without response, so later on he underwent repeated Botox injections with remarkable improvement. 

## 4. Conclusion

This paper supports the use of Botox in the treatment of focal dystonia and myokymia in patients with LSCS.
